# Editorial: Excessive and problematic smartphone usage, volume II

**DOI:** 10.3389/fpsyt.2023.1181652

**Published:** 2023-04-27

**Authors:** Aviv M. Weinstein, Kristiana Siste

**Affiliations:** ^1^Psychology and Behavioral Science, Ariel University, Ari'el, Israel; ^2^Department of Psychiatry, Faculty of Medicine, Universitas Indonesia, Dr. Cipto Mangunkusumo General Hospital, Jakarta, Indonesia

**Keywords:** excessive, smartphone, use, problematic smartphone use, social media use

The use of smartphones has increased drastically over the last decade and has become a part of everyday human life. Smartphones bring convenience and comfort to many people, for example, in terms of shopping, studying, working, and communicating online. However, excessive smartphone use negatively impacts various aspects of human life, for example, by increasing anxiety and depression. Many controversies exist about whether excessive smartphone use can be stated as part of behavioral addiction. Problematic or excessive smartphone use has been related to the characteristics of behavioral addiction such as use despite of harmful consequences, tolerance and withdrawal. Finally, problematic smartphone use can cause interference with work, social, and daily life functions. It has demanded special care since it has been associated with cognitive, emotional and social impairments and some medical harm especially in sleep quality. Six papers in this Research Topic have studied the various psychological, behavioral, and health issues associated with Problematic Smartphones Use (PSU).

Previous research has investigated the core symptoms of PSU. However, most studies have used a cross-sectional design that cannot make an inference on causality. Huang et al. have used a longitudinal design to investigate the association between symptoms of PSU and its development. Two thousand one hundred ninety-one adolescents were followed for 3 years for PSU symptoms. The results have indicated that symptoms of PSU like loss of control affect their longitudinal development. Furthermore, losing control and being caught in the loop had the highest prediction over other symptoms. These findings confirm the key roles of core symptoms in developing PSU and suggest that these should be addressed during treatment.

Previous studies have shown that individuals with PSU have difficulties in emotion regulation, but the effectiveness of their emotion regulation strategies has yet to be investigated. Liu et al. have instructed individuals with PSU to handle negative emotions by using cognitive and emotional techniques. They have reported that individuals with PSU showed faster responses to negative affect indicating impaired emotion regulation techniques.

Kim et al. have reported a study on mental health based on a survey among adolescents with smartphone dependence. Four hundred eighty-two respondents were included based on their experience of smartphone dependence, and 241 participants were included as a high-dependent group compared with 241 age- and gender-matched control participants. They have found that mental health measures (unhappy, stressed, and lonely) have increased the likelihood of having smartphone dependence. In contrast, impaired sleep quality has reduced the likelihood of having smartphone dependence. These findings have important clinical implications for preventing and treating mental health problems in adolescents with PSU using methods for self-control and developing desirable life habits.

Two studies have investigated the neuro-biological effects of PSU. Kwon et al. have reported a study of functional connectivity in problematic smartphone users in fMRI.

They have found greater functional connectivity of the dorsal anterior cingulate cortex (dACC) with the ventral attention network (VAN) and the Default Mode Network in problematic smartphone users. This functional connectivity was associated with smartphone addiction ratings among individuals with PSU. These findings suggest that increased attentional processing plays an essential role in PSU.

Xiang et al. have used the Stroop task to measure inhibitory control mechanisms with near-infrared spectroscopy (fNIRS). The PSU group have shown greater Stroop interference indicating impaired inhibitory control correlating with lower cortical activation in the left DLPFC. Finally, Yang et al. have reported a study of sleep disorder patients undergoing acupuncture treatment for seven days. They have found that saliva measures of major metabolites were reduced after treatment, indicating the potential usefulness of this treatment for sleep disorder.

In conclusion, we have collected studies showing that PSU symptoms can predict future development of the disorder. PSU is also associated with impaired cognitive-emotional regulation, increased attentional processing mediated by the dorsal anterior cingulate cortex, impaired inhibitory control, and lower cortical activation in the left DLPFC. There is also preliminary evidence that acupuncture can ameliorate sleep disorders in individuals with PSU. These studies complement our previous Research Topic, which included studies showing impaired cognitive function, emotional and medical problems, and especially sleep disturbances in PSU. These studies have important clinical implications for our understanding and treatment of PSU.

## Author contributions

Both authors listed have made a substantial, direct, and intellectual contribution to the work and approved it for publication.

